# Liver-specific deletion of IGF2 mRNA binding protein-2/IMP2 reduces hepatic fatty acid oxidation and increases hepatic triglyceride accumulation

**DOI:** 10.1074/jbc.RA119.008778

**Published:** 2019-06-17

**Authors:** Laura Regué, Liliana Minichiello, Joseph Avruch, Ning Dai

**Affiliations:** ‡ Department of Molecular Biology, Massachusetts General Hospital, Boston, MA 02114; § Diabetes Unit of the Medical Services, Massachusetts General Hospital, Boston, MA 02114; ¶ Department of Medicine, Harvard Medical School, Boston, MA 02115; ‖ Department of Pharmacology, University of Oxford, Oxford OX1 3QT, United Kingdom

**Keywords:** liver metabolism, lipid metabolism, fatty acid oxidation, RNA binding protein, hepatocyte, obesity, carnitine palmitoyltransferase 1A (CPT-1A), IGF2 mRNA binding protein 2, IGF2BP2, IMP2, peroxisome proliferator–activated receptor α (PPARα)

## Abstract

Insulin-like growth factor 2 mRNA-binding proteins 1–3 (IGF2BP1–3, also known as IMP1–3) contribute to the regulation of RNAs in a transcriptome-specific context. Global deletion of the mRNA-binding protein insulin-like growth factor 2 mRNA-binding protein 2 (IGF2BP2 or IMP2) in mice causes resistance to obesity and fatty liver induced by a high-fat diet (HFD), whereas liver-specific IMP2 overexpression results in steatosis. To better understand the role of IMP2 in hepatic triglyceride metabolism, here we crossed mice expressing albumin-Cre with mice bearing a floxed *Imp2* gene to generate hepatocyte-specific IMP2 knockout (LIMP2 KO) mice. Unexpectedly, the livers of LIMP2 KO mice fed an HFD accumulated more triglyceride. Although hepatocyte-specific IMP2 deletion did not alter lipogenic gene expression, it substantially decreased the levels of the IMP2 client mRNAs encoding carnitine palmitoyltransferase 1A (CPT1A) and peroxisome proliferator–activated receptor α (PPARα). This decrease was associated with their more rapid turnover and accompanied by significantly diminished rates of palmitate oxidation by isolated hepatocytes and liver mitochondria. HFD-fed control and LIMP2 KO mice maintained a similar glucose tolerance and insulin sensitivity up to 6 months; however, by 6 months, blood glucose and serum triglycerides in LIMP2 KO mice were modestly elevated but without evidence of liver damage. In conclusion, hepatocyte-specific IMP2 deficiency promotes modest diet-induced fatty liver by impairing fatty acid oxidation through increased degradation of the IMP2 client mRNAs *PPAR*α and *CPT1A*. This finding indicates that the previously observed marked protection against fatty liver conferred by global IMP2 deficiency in mice is entirely due to their reduced adiposity.

## Introduction

IGF2 mRNA binding proteins 1–3 (IGF2BP1–3, also called IMP1–3) participate in regulation of the RNA life cycle in a transcriptome-specific manner (1, 2). Each IGF2 mRNA binding protein (IMP)^4^ is able to bind diverse RNA species, including long noncoding RNAs and mRNAs, to control their splicing (especially IMP1), transport, translation, and stability. All three IMPs are expressed coordinately in the mouse embryo starting at ∼E10.5 and peaking at ∼E12.5. The expression of *Imp1* and *Imp3* is largely extinguished after birth, whereas *Imp2* is widely expressed postnatally (3, 4).

Genome-wide association studies of many populations have identified SNPs in the second intron of the human *IMP2* gene that occur in excess in individuals with type 2 diabetes (5–7). Because little was known about the biological functions of IMP2 *in vivo*, we characterized mice with global IMP2 deficiency (8). In contrast to *Imp1*-null mice, which are born small and show ∼50% mortality at postnatal day 3 (9), *Imp2*-null mice appear normal at birth, and their body weight is similar to WT littermates until weaning; thereafter, however, *Imp2*-null mice gain less weight because of slower accumulation of both lean and fat mass. The reduced skeletal muscle mass seen with global IMP2 deficiency is due to a myocyte-intrinsic diminution in protein synthesis, attributable in part to diminished autocrine IGF2 production (10). The lesser fat mass of *Imp2*-null mice is especially marked on a high-fat diet and is accompanied by reduced circulating lipids, markedly less liver triglyceride accumulation, and much better glucose tolerance and insulin sensitivity. Regarding the mechanism(s) underlying resistance of mice with global IMP2 deficiency to fatty liver, the relative contribution of reduced FFA delivery *versus* altered hepatocyte triglyceride metabolism is not known. Notably, transgenic overexpression of IMP2 in mouse liver results in increased triglyceride deposition (11), perhaps in part through up-regulation of hepatocyte IGF2 expression (12, 13).

In addition to their altered metabolism, *Imp2*-null mice are long-lived and exhibit fewer malignancies at an advanced age (8). Considerable evidence implicates human IMP2 as a tumor promoter that is overexpressed in many common human cancers and associated with an adverse outcome (14–16). For example, the 62-kDa variant of the IMP2 polypeptide has been identified as an autoantigen in human hepatocellular carcinoma (HCC); its expression is low in normal adult liver but high in hepatocellular carcinoma nodules and fetal liver (17). In mice with liver-specific IMP2 overexpression, treatment with diethylnitrosamine is accompanied by an earlier onset and accelerated progression of hepatic tumorigenesis than in diethylnitrosamine-treated controls (18). It is unclear whether the steatosis accompanying hepatic IMP2 overexpression contributes to the pro-tumorigenic phenotype.

The finding that IMP2 hepatic overexpression results in steatosis whereas global IMP2 deficiency is accompanied by a marked reduction in hepatic triglyceride suggested that IMP2 may favor liver triglyceride deposition. To examine the role of endogenous IMP2 in hepatocyte triglyceride balance, *Imp2ff* mice were crossed with mice expressing cre recombinase driven by the albumin promoter. Liver-specific *Imp2ff* knockout (LIMP2 KO) mice exhibit a marked loss of IMP2 expression in postnatal liver. When consuming a high-fat diet, LIMP2 KO mice unexpectedly exhibited increased hepatic triglyceride accumulation and, ultimately, elevated blood triglyceride levels. We demonstrate that this results from a reduction in hepatic fatty acid oxidation caused by more rapid turnover and decreased abundance of the IMP2 client mRNAs encoding the CPT1A and PPARα polypeptides.

## Results

### Generation of hepatocyte-specific IMP2 knockout mice

**Figure 1.**
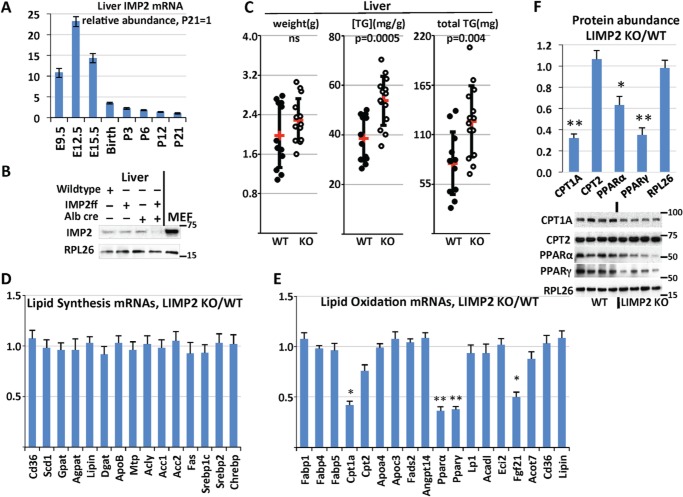
**Selective deletion of IMP2 from mouse liver causes increased triglyceride deposition.** *A*, relative liver IMP2 mRNA abundance during development. Livers were harvested from three WT male mice at the indicated times. The abundance of IMP2 mRNA was determined by QPCR, normalized to the level of 18S RNA, and divided by the IMP2 mRNA at postnatal day 21. *B*, abundance of IMP2 polypeptide in the livers of C57/Bl6j, *Imp2ff*, Alb-Cre^+/−^, and *Imp2ff*/Alb-Cre^+/−^ mice. Livers were taken from three males of each genotype at 8 weeks age on the HFD. The numbers on the *left* in the blot in this and subsequent figures indicates the *M*_r_ in kilodaltons of nearby molecular mass markers. *MEF*, mouse embryonic fibroblast. *C*, liver triglyceride (*TG*) levels in *Imp2ff* and *Imp2ff*/Alb-Cre^+/−^ mice. Male mice, 12 *Imp2ff and* 14 *Imp2ff*/Alb-Cre^+/−^, 30 weeks of age, on the HFD from 4 weeks; mean values are indicated in *red. D* and *E*, relative abundance of liver mRNAs concerned with neutral lipid synthesis (*D*) and catabolism (*E*). RNA was extracted from livers of four pairs of *Imp2ff* and *Imp2ff*/Alb-Cre^+/−^ mice fed the HFD at 30 weeks of age, and the relative abundance of the mRNAs indicated was determined by QPCR. Oligonucleotides are listed in Table S1. *F*, immunoblot of *Imp2ff* and *Imp2ff*/Alb-Cre^+/−^ liver extracts. HFD-fed male mice, 30 weeks of age. Antibodies are listed in Table S1. *, *p* < 0.05; **, *p* < 0.01.

During mouse development, expression of IMP2 mRNA in the liver peaks around E12.5 and diminish sharply after birth (Fig. 1*A*); however, at E12.5, the liver is primarily a hematopoietic organ, and hepatoblasts are relatively few (22). Liver IMP2 polypeptide expression in WT and LIMP2 KO mice was examined by immunoblot at 2 months age; liver IMP2 protein was greatly reduced in homozygous LIMP2 KO mice (Fig. 1*B*). Additional immunoblot studies of LIMP2 KO with tissues from fat, muscle, and brain showed no significant difference in IMP2 protein level compared with control animals, confirming the tissue specificity of Cre expression (data not shown).

### Deletion of Hepatocyte IMP2 promotes triglyceride deposition

Mice were placed on a high-fat diet (HFD) from weaning. No significant differences in body weight or body composition of WT and LIMP2 KO mice were observed, either at weaning or after 30 weeks on normal chow or on the high-fat diet (Fig. S1). Although hepatic triglyceride content in WT and LIMP2 KO male mice at 10 weeks age is similar (Fig. S2*A*), by 30 weeks of age, the livers of LIMP2 KO male mice on the HFD contained ∼60% more triglyceride than control livers (Fig. 1*C*). In female mice, although a tendency toward higher triglyceride levels was observed in LIMP2 KO livers of mice on both normal chow (Fig. S2*B*) and the HFD (Fig. S2*C*), liver triglycerides were highly variable and not statistically significant. Thus, hepatocyte expression of IMP2 is protective against development of fatty liver in male mice, whereas the effect in females remains to be determined. Subsequent studies focused on male mice.

### Down-regulation of lipid oxidation genes in the livers of LIMP2 KO mice

To explore the mechanisms underlying increased lipid accumulation in the livers of male LIMP2 KO mice, we performed real-time PCR for mice fed a HFD at 30 weeks. The analysis revealed that albumin-Cre–mediated deletion of the *Imp2* gene in the liver had no effect on the relative abundance of a cohort on mRNAs encoding elements important for lipogenesis (ACC1, ACC2, FAS, ACLY, SREBP1c, SREBP2, and ChREBP) and triglyceride synthesis (CD36, SCD1, GPAT, AGPAT, Lipin, DGAT, ApoB, and MTP), apart from a 62% reduction in PPARγ mRNA (Fig. 1*D*). By contrast, IMP2 deletion resulted in significant down-regulation of several mRNAs encoding elements critical for fatty acid oxidation: a 60% decrease in CPT1A mRNA, a 64% decrease in PPARα mRNA, a 50% decrease in FGF21 mRNA, and a nonsignificant 24% decrease in CPT2 mRNA (Fig. 1*E*). Western blots of the PPARα, PPARγ, and Cpt1A polypeptides confirmed the reduced abundance of these polypeptides, whereas CPT2 was unaltered (Fig. 1*F*). Altogether, these data suggest that IMP2 promotes lipid oxidation without affecting neutral lipid synthesis.

### Liver IMP2 deficiency results in diminished oxidation of palmitate

**Figure 2.**
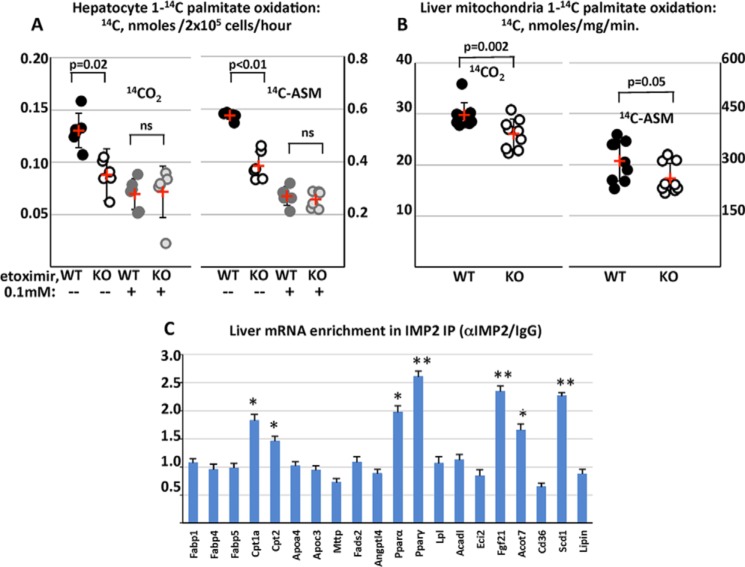
**IMP2 inactivation diminishes palmitate oxidation by hepatocytes and liver mitochondria and binds mRNAs encoding mediators of fatty acid oxidation.** *A*, [1-C^14^]palmitate oxidation by hepatocytes from *Imp2ff* and *Imp2ff*/Alb-Cre^+/−^ mice. Hepatocytes were isolated from six pairs of *Imp2ff* and *Imp2ff*/Alb-Cre^+/−^ male mice 6 weeks of age, 2 weeks on the HFD, and assayed in triplicate for their ability to oxidize [1-C^14^]palmitate with or without etoximir (0.1 mm). Mean values are indicated in *red. ns*, *p* > 0.05. *B*, [1-C^14^]palmitate oxidation by mitochondrial fractions from *Imp2ff* and *Imp2ff*/Alb-Cre^+/−^ livers. Mitochondria were isolated from the livers of nine pairs of *Imp2ff* and *Imp2ff*/Alb-Cre^+/−^ male mice 6 weeks age, 2 weeks on the HFD, and assayed in triplicate for their ability to oxidize [1-C^14^]palmitate. *C*, liver RNAs enriched in an IMP2 IP. Extracts from the livers of three pairs of 30-week-old HFD-fed male *Imp2ff* and 8 *Imp2ff*/Alb-Cre^+/−^ mice were incubated with anti-IMP2 or nonimmune IgG. RNA was extracted from the washed IPs, and the indicated mRNAs were quantified by QPCR. The ratio of mRNA abundance in IMP2 IP/nonimmune IP is shown. *, *p* < 0.05; **, *p* < 0.01.

The ability of primary mouse hepatocytes (Fig. 2*A*) and liver mitochondria (Fig. 2*B*), prepared from the livers of 6-week-old male LIMP2 KO and WT mice, to carry out oxidation of [1-^14^C]palmitate was determined. In hepatocytes, generation of ^14^CO_2_ and ^14^C-labeled acid-soluble metabolites (ASM) from [1-^14^C]palmitate by LIMP2 KO cells was 33% and 36% less than in WT hepatocytes, whereas essentially identical levels of ^14^CO_2_ and [^14^C]ASM were generated from LIMP2 KO and WT hepatocytes when they were pretreated briefly with the CPT-1 inhibitor etoximir (Fig. 2*A*). Thus, hepatocyte IMP2 deficiency reduces mitochondrial fatty acid oxidation by intact cells. This was confirmed by an assay of [1-^14^C]palmitate oxidation by mitochondrial preparations isolated from livers of 6-week-old male LIMP2 KO and WT mice. Generation of ^14^CO_2_ by LIMP2 KO mitochondria was reduced by 15% (*p* < 0.002) and that of [^14^C]ASM by 18.8% (*p* = 0.05) compared with isolates from WT livers (Fig. 2*B*).

### IMP2 binds and stabilizes CPT-1A and PPARα mRNAs and promotes CPT-1A mRNA translation

IMP2 is an RNA-binding protein that can regulate the life cycle of client mRNAs through altered transport, stability, and translation. To identify IMP2 mRNA clients relevant to fat metabolism in the liver, immunoprecipitates (IPs) were prepared from extracts of hepatocytes isolated from 30-week-old mice using anti-IMP2 and nonimmune IgG; RNA extracted from the IPs was quantitated by RT-PCR. Compared with IPs obtained with nonimmune IgG, the IMP2 IP was substantially enriched in mRNAs encoding PPARα, PPARγ, CPT1A, FGF21, ACOT7, and SCD1, indicating that these are IMP2 clients, whereas CPT2 enrichment (1.46-fold), although statistically significant, was marginal (Fig. 2*C*).

**Figure 3.**
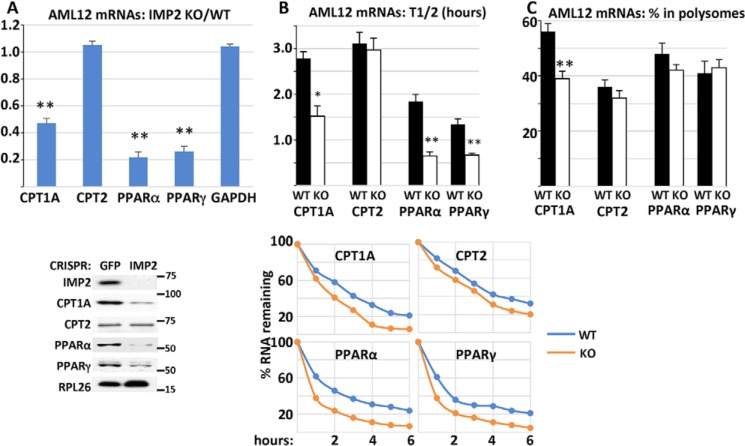
**The effect of *Imp2* inactivation in AML12 hepatocytes on the overall abundance, half-life, and polysomal abundance of selected RNAs.** *A*, the effects of CRISPR-catalyzed *Imp2* inactivation in AML12 hepatocytes on the abundance of selected mRNAs and polypeptides. RNA was extracted from AML12 hepatocytes transduced with CRISPR reagents directed at *Imp2* or GFP, and the abundance of the indicated mRNAs was estimated by QPCR. The ratio of IMP2 CRISPR/GFP CRISPR is shown. *Bottom panel*, immunoblots of IMP2 and other polypeptides. *B*, mRNA half-life in IMP2 CRISPR and GFP CRISPR AML12 cells. Cells were treated with actinomycin (1 μm) for 12 h. RNA was extracted at *t* = 0 and every 2 h thereafter, and the abundance of the indicated mRNAs was determined by QPCR. The rate of decline was plotted by least squares using the first three time points shown in the *bottom panel*, and the time to 50% decrease of the initial value is shown in the *top panel. C*, the percentage of mRNA residing in polysomes in IMP2 CRISPR/GFP CRISPR AML12 cells. Total RNA and post-mitochondrial extracts were prepared from equal numbers of rapidly growing cells. The post-mitochondrial extracts were subjected to density gradient centrifugation. Total RNA and RNA from the pooled polysomal fraction of the gradients were quantified by QPCR, and the ratio of polysomal RNA/total RNA × 100 is shown. *, *p* < 0.05; **, *p* < 0.01.

We next sought to understand whether IMP2 regulates the stability and/or translation of its client mRNAs in the lipid oxidation pathway. We used CRISPR to delete IMP2 from AML12 mouse hepatocytes (23) and compared these IMP2 CRISPR AML12 cells with GFP CRISPR control AML12 cells. Compared with CRISPR-GFP control AML12 cells, IMP2 CRISPR AML12 cells exhibit a 53% reduction in the abundance of CPT-1A mRNA (Fig. 3*A*), accompanied by a significantly shortened mRNA half-life (Fig. 3*B*, 2.78 h *versus* 1.52 h) and reduced mRNA polysomal abundance (Fig. 3*C*, 56% *versus* 39%). CRISPR deletion of IMP2 from AML12 cells also caused a substantial reduction in CPT-1A polypeptide abundance (Fig. 3*A*, *bottom panel*). Thus, the 68% decrease in CPT-1A polypeptide abundance in the IMP2-deficient liver is probably in part due to reduced CPT-1A mRNA stability and reduced translational efficiency. The abundance of PPARα mRNA in IMP2 CRISPR AML12 cells was reduced by 78% (Fig. 3*A*) with a shortened *t*_½_ from 1.84 h to 0.65 h compared with that for PPARα mRNA in GFP CRISPR AML12 cells (Fig. 3*B*); PPARα mRNA polysomal abundance was not altered (Fig. 3*C*). Thus, IMP2 deficiency reduces PPARα mRNA stability but not translation. The decreased level of PPARα in IMP2 CRISPR cells may contribute an element of reduced *Cpt1a* transcription (24). Although CPT-2 mRNA is a candidate IMP2 client, CPT-2 polypeptide abundance in the liver is not altered by IMP2 deficiency in the liver (Fig. 1*F*, *bottom panel*) or in AML cells (Fig. 3*A*, *bottom panel*), nor were there differences in CPT2 mRNA abundance, *t*_½_, or polysomal abundance (Fig. 3, *A–C*) in IMP2 CRISPR AML12 cells.

### LIMP2 KO mice develop hypertriglyceridemia and slight hyperglycemia by 30 weeks of age

**Figure 4.**
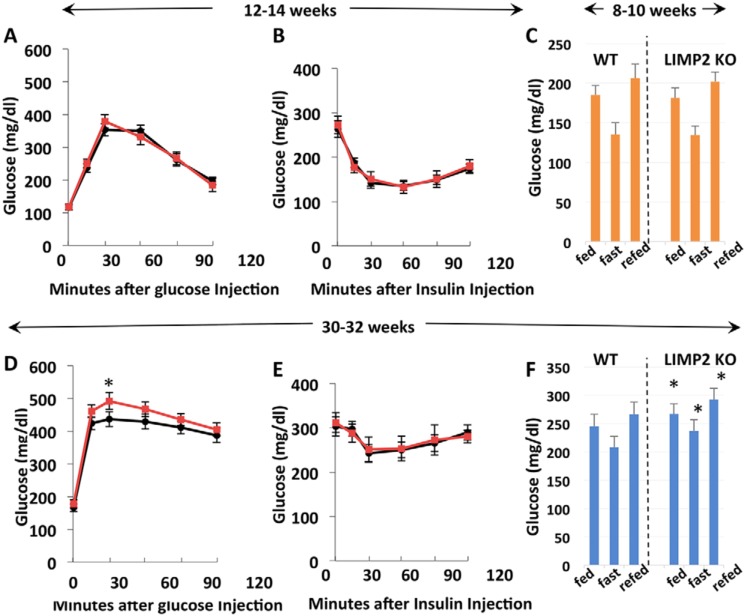
**Blood glucose levels, glucose tolerance, and insulin tolerance in *Imp2ff* and *Imp2ff*/Alb-Cre^+/−^ male mice fed a high-fat diet.** *A* and *D*, glucose tolerance in eight pairs at 12 (*A*) and 30 weeks of age (*D*). *B* and *E*, insulin tolerance in eight pairs at 14 (*B*) and 32 weeks of age (*E*). *C* and *F*, blood glucose response to fasting and refeeding. Blood glucose was measured before withdrawal of chow (*fed*), 16 h after food deprivation (*fast*), and 2 h after return of access to food (*refed*) at 8–10 (*C*) and 33 weeks of age (*F*). *, *p* < 0.05.

At 10 weeks of age, the levels of blood glucose, insulin, glucagon, leptin, cholesterol, FFA, and triglycerides of control and LIMP2 KO mice were similar (Table 1). In addition, the glucose tolerance and insulin sensitivity of LIMP2 KO and WT mice did not differ at 12 and 30 weeks and 14 and 32 weeks of age, respectively (Fig. 4, *A–E*). However, at 30–32 weeks of age, LIMP2 KO mice displayed increased serum triglycerides (Table 1), and blood glucose, whether fed, fasting, or fast-refeeding, was slightly but significantly higher in LIMP2 KO mice (Fig. 4*F*). At this time, serum ALT and AST levels were not elevated in the LIMP2 KO mice (Table 1), nor was their liver histology (H&E stain, Fig. S3) visibly different from WT littermates.

**Table 1:** **Some serum values in WT and LIMP2 KO mice** Values in bold highlight statistically significant differences.

Genotype	8–10 weeks of age			28–30 weeks of age		
	WT	LIMP2 KO	*p* value	WT	LIMP2 KO	*p* value
**Males, 6 pairs, HFD, 6-h fast**						
Blood glucose (mg/dl)	127.6	128.3	0.48	218.4	236.7	**0.02**
Serum insulin (ng/dl)	0.43	0.41	0.52	5.1	4.9	0.56
Serum glucagon (pg/ml)	0.01	0.01	0.65	0.06	0.05	0.26
Serum leptin (ng/ml)	0.9	0.8	0.43	7.2	7.3	0.83
Serum cholesterol (mg/dl)	86.4	88.2	0.11	172.4	210.4	0.06
Serum triglyceride(mg/dl)	26.2	27.8	0.26	120.4	153.8	**0.03**
Serum FFA (mmol/I)	0.2	0.2	0.82	1.2	1.1	0.68
**Males, 4 pairs, HFD, 6-h fast**						
Alanine aminotransferase (normal = 10–190 units/liter)				69.3	53.8	0.28
Aspartate aminotransferase (normal = 10–380 units/liter)				119.3	94.4	0.28

## Discussion

The primary finding reported here is that elimination of the IGF2 mRNA-binding protein 2/IMP2 from adult mouse liver is accompanied by increased intrahepatic triglyceride deposition in mice fed a high-fat diet. This outcome was unexpected because transgenic overexpression of IMP2 in hepatocytes is accompanied by increased triglyceride deposition (11), and global elimination of IMP2 confers strong protection against fatty liver in mice fed a high-fat diet (8). We have no direct information concerning the mechanisms by which transgenic IMP2 overexpression causes hepatic steatosis; however, a plausible inference is that transgenic overexpression of IMP2 in hepatocytes modifies the abundance of client RNAs, including IGF2 mRNA (13) and perhaps including some mRNAs not recruited by the normally low levels of IMP2 in adult hepatocytes. Although the latter might be considered an “artifact” of IMP2 overexpression, the cohort of IMP2 RNA clients does vary in a tissue-specific manner, determined both by the abundance of IMP2 and of its candidate client RNAs. Regarding the protective effect of global IMP2 deficiency on liver triglyceride deposition in mice fed a HFD, the present results indicate strongly that this protection is entirely due to the lesser mass of white adipose tissue in *Imp2*-null mice and is probably offset slightly because of the diminished fatty acid oxidation of the IMP2-deficient liver. A caveat, however, is that we have not characterized lipid metabolism in hepatocytes derived from mice with germline *Imp2* inactivation. It remains conceivable, therefore, that loss of IMP2 in endoderm or liver bud precursors might reprogram hepatocyte triglyceride metabolism in a manner distinct from that engendered by deletion using albumin-cre.

Regarding the mechanism by which IMP2 deficiency in the adult liver causes increased triglyceride deposition, we show that the mRNAs encoding PPARα, PPARγ, CPT-1A, FGF21, and SCD1 are IMP2 clients, and elimination of IMP2 was accompanied by substantially reduced levels of PPARα, PPARγ, and CPT1A mRNAs because of a 2-fold or more increased rate of mRNA turnover; the corresponding polypeptide levels were comparably reduced. Notably, expression of the *Cpt1A* gene was strongly up-regulated by PPARα, so that the somewhat greater reduction of CPT-1A mRNA consequent to *Imp2* inactivation may reflect the loss of PPARα-stimulated *Cpt1A* transcription as well (24). Direct measurement of [1-^14^C]palmitate oxidation by intact hepatocytes and liver mitochondria demonstrated a modest reduction by those isolated from IMP2-deficient liver. In contrast, the mRNAs of all lipogenic genes examined (including SCD1) were unaltered in IMP2-deficient liver. This is somewhat surprising given the substantial drop in PPARγ in LIMP2 KO liver, which is generally considered to drive lipogenesis (25).

In summary, IMP2 binds and stabilizes the mRNAs encoding the critical regulators of hepatic fatty acid oxidation, PPARα and CPT-1A; loss of IMP2 diminishes the abundance of those mRNAs, resulting in reduced mitochondrial fatty acid oxidation. Over time, mice with hepatic IMP2 deficiency fed a high-fat diet show a modest, progressive accumulation of hepatic triglycerides beyond that of HFD-fed controls, ultimately reflected in elevated circulating triglycerides and mildly elevated blood glucose. Notably, however, evidence of liver damage is lacking, at least at 6 months age. The proclivity to increased hepatic triglyceride deposition caused by hepatocyte-selective IMP2 deficiency is entirely masked in mice with global IMP2 deficiency by their marked reduction in white adipose mass. For hepatocyte IMP2 deficiency to contribute to type 2 diabetes risk, the intronic SNPs in the human IMP2 gene associated with type 2 diabetes would have to cause a profound hepatocyte-selective decrease in IMP2 expression, an outcome that appears highly unlikely. Moreover, recent evidence indicates that the diabetogenic risk conferred by the intronic SNPs is attributable to a reduction of IMP2 abundance in β cells (26).

## Materials and methods

### Animal studies

Generation of floxed-IMP2 mice is described in Ref. 10. The albumin-cre mice were purchased from The Jackson Laboratory (stock no. 003574). Hemizygous Alb-Cre transgenic mice were crossed with mice homozygous for a floxed Imp2 allele (*Imp2ff*), the latter backcrossed onto the C57Bl/6J background for 12 generations. F1 pups with the Alb-Cre^+/−^/*Imp2f*+ haplotype were crossed again with *Imp2ff* mice, generating F2 pups with four different genotypes: Alb-Cre^+/−^/*Imp2ff* (hereafter called homozygous LIMP2 KO), *Imp2ff* (referred to as WT), Alb-Cre^+/−^/*Imp2f*+, and *Imp2f*+; the latter two genotypes were not used in this study. All animal procedures were approved by the Institutional Animal Care and Use Committee of Massachusetts General Hospital. Mice were maintained on the C57Bl/6J background in a specific pathogen–free facility with a 12:12 h light:dark cycle. After weaning at 4 weeks, they were fed irradiated chow (Prolab 5P75 Isopro 3000, 5% crude fat, PMI Nutrition International) or a high-fat diet (D12492i, 60 kcal percent fat; Research Diets Inc.). Additional aspects of animal care were as described in Ref. 10.

### Glucose and insulin tolerance tests

Mice were fasted overnight (16 h). Twenty percent D-glucose (Sigma) (1 g/kg of body weight) was administered by intraperitoneal injection. 0, 20, 40, 60, and 120 min after administration, blood was collected by tail vein bleeding. Glucose levels were measured with a One Touch Ultra AlphaTrak2 glucometer (Zoetis, Parsippany, NJ). For insulin tolerance tests, mice fed the high-fat diet were fasted for 5 h. Human insulin (Eli Lilly, 0.75 units/kg) was injected intraperitoneally. Blood was drawn from the tail vein 0, 20, 40, 60, and 90 min after injection, and glucose levels were measured as above.

### Serum analyses

Blood was collected into EDTA-coated tubes (Sarstedt, Newton, NC). Serum was separated by centrifugation at 4 °C, frozen in liquid nitrogen, and assayed by the Vanderbilt University Mouse Metabolic Phenotyping Center.

### Liver lipid analyses

Livers were collected, weighed, and snap-frozen in liquid nitrogen. Liver triglyceride content was measured by the Vanderbilt Hormone Assay and Analytical Service Core.

### Real-time PCR

Total liver RNA was extracted directly using the Qiagen RNase kit, and 1 μg was used for complementary DNA preparation with random hexamer primers using Super Script III reverse transcriptase (Invitrogen). Steady-state mRNA expression was measured by quantitative real-time PCR using SYBR Green Master Mix (Bio-Rad) with a CFX96 real-time PCR system (Bio-Rad). Primer sequences for real-time PCRs are listed in Table S1.

### IMP2-associated RNAs

Livers were extracted using a tissue homogenizer (Qiagen) for 10 min in ice-cold lysis buffer (140 mm KCl, 1.5 mm MgCl_2_, 20 mm Tris-HCl (pH 7.4), 0.5% Nonidet P-40, 0.5 mm DTT, 1 unit/μl RNase inhibitor, and one complete EDTA-free protease inhibitor mixture tablet). The lysates were centrifuged for 10 min at 14,000 rpm, and the supernatant was transferred to a fresh 1.5-ml tube. Total protein was measured by Bradford assay, and 5 mg of cytoplasmic lysate protein was subjected to immunoprecipitation as in Ref. 8. Lysates were incubated with 500 μl of protein A magnetic Dynabeads precoated with IMP2 antibody or nonimmune IgG and incubated for 6 h at 4 °C with rotation. Dynabeads were washed extensively with lysis buffer five times and digested with DNase I and protease K. RNA was extracted with phenol/chloroform and precipitated with ethanol. Real-time PCR was performed to examine RNAs associated with cytoplasmic IMP2.

### Immunoblotting

Proteins were extracted from the livers of HFD-fed WT male mice 6–8 weeks of age. Livers were homogenized using a tissue homogenizer (Qiagen) in ice-cold buffer (20 mm Tris (pH 7.5), 2.7 mm KCl, 1 mm MgCl_2_, 1% Triton X-100, 10% (w/v) glycerol, 1 mm EDTA, and 1 mm DTT) supplemented with protease (Thermo Scientific) and phosphatase inhibitor mixture (Millipore). Samples were then centrifuged at 13,000 rpm for 10 min at 4 °C, and the supernatants were collected. The protein content of the supernatant was determined using a BCA assay (Thermo Scientific). 50 μg of each sample was loaded for Western blotting. Proteins were resolved on a 4% to 12% gradient SDS BisTris gel (Invitrogen). The IMP2 antibody is described in Ref. 4, and sources of other antibodies are shown in Table S1. All commercial antibodies were used at 1:1000 dilution, with specificity validated by the vendors.

### Fatty acid oxidation by liver mitochondria

Liver mitochondria were isolated from 6-week-old male mice as described in Ref. 19. The protocol for the fatty acid oxidation assay was adapted from Ref. 20. *Imp2ff* and Alb-cre^+/−^/*Imp2ff* mitochondria were incubated in triplicate for 30 min at 37 °C in a reaction mixture with 0.7% FFA-poor BSA, 500 μm palmitate, and 1 μCi [^14^C]palmitate (21). The reaction mixtures were then transferred to a new tube containing 1 m cold perchloric acid and, in the cap, a paper disc saturated with 1 m NaOH. After closing the cap, the tubes were incubated for 1 h at 37 °C. [^14^C]radioactivity contained in the paper and the acidic fraction was measured by liquid scintillation counting.

### Isolation of mouse hepatocytes

Six-week-old *Imp2ff* and Alb-cre^+/−^/*Imp2ff* male mice fed the HFD were anesthetized, and perfusion was performed through the portal vein with 20 ml of perfusion buffer (Krebs–Ringer with 3.6 mg/ml glucose and 1 m CaCl_2_), followed by 30 ml of digestion buffer (perfusion buffer with 0.66 mg/ml collagenase I, Worthington, Lakewood, NJ) at 37 °C. The liver was aseptically removed to a sterile 10-cm cell culture dish containing 10 ml of cold perfusion buffer. The excised liver was cut, and hepatocytes were dispersed by aspiration with a large-bore pipette, followed by filtration through a 70-μm cell strainer (Fisher) into a 50-ml centrifuge tube and centrifugation at 50 × *g* for 2 min at 4 °C. Cells were then suspended with cold wash medium (Williams medium, Gibco) with 1.06 g/ml Percoll, followed by centrifugation at 50 × *g* for 10 min at 4 °C to enrich for viable hepatocytes. Pelleted cells were then washed three times in wash medium and resuspended in 15 ml of complete Williams medium E (2 mm l-glutamine, 20 units/ml penicillin, 20 μg/ml streptomycin, 1 nm insulin, 1 nm dexamethasone, and 1 mm carnitine) supplemented with 10% FBS. After the viability was determined by trypan blue staining, the hepatocytes were plated in 6-well culture plates precoated with collagen (Collagen I Cellware, Corning, Kennebunk, ME) and cultured at 37 °C and 5% CO_2_. After 4 h, the medium was changed into complete Williams medium E without FBS, and the cells were cultured for 10 h before the assay.

### Fatty acid oxidation by primary mouse hepatocytes

The mouse hepatocyte fatty acid oxidation assay was adapted from Ref. 20. Primary hepatocytes (2 × 10^5^/well) were incubated with etomoxir (100 μm) or vehicle at 37 °C 5% CO_2_. After 1 h, cells were washed with assay medium (DMEM, 1 nm insulin, 1 nm dexamethasone, and 1 mm carnitine), followed by incubation for 3 h at 37 °C with assay medium containing 0.3% BSA and 100 μm [1-^14^C]palmitate (with or without 100 μm etomoxir). The reaction was stopped by transfer of the culture supernatant to 1 m perchloric acid vials with ^14^CO_2_ traps. The tubes were incubated for 1 h at 37 °C. [^14^C]cpm contained in the paper and the acidic fraction was measured by liquid scintillation counting.

### CRISPR of IMP2 from AML12 cells

AML12, a nontransformed mouse hepatocyte cell line derived from mice transgenic for transforming growth factor α, were obtained from the ATCC and cultured in Dulbecco's modified Eagle's medium supplemented with F12 (DMEM-F12; Life Technologies) containing 11 mm glucose. CRISPR-catalyzed inactivation of Imp2 was carried out as in Ref. 16 using CRISPR-Cas reagents obtained from Addgene (U6-Chimeric_BB-CBh-hSpCas9). Small guide RNAs, selected for minimal predicted off-target mutagenesis, were designed using CRISPR design software available online from the Zhang lab at MIT (m*Imp2*_CRISPR 1, aaacTCAAGATTTCCTACATCCCC; m*Imp2*_CRISPR 2, caccGGGAACAAGGCCACGGCCCC).

### Polysome isolation

AML12 cells were rinsed with PBS and lysed with ice-cold buffer (140 mm KCl, 1.5 mm MgCl_2_, 20 mm Tris-HCl (pH 7.4), 0.5% Nonidet P-40, 0.5 mm DTT, 1 unit/μl RNase inhibitor, and one complete EDTA-free protease inhibitor mixture tablet) with 150 mg/ml cycloheximide, 1000 units/ml RNase inhibitor (Roche Diagnostics), and 40 mm vanadyl–ribonucleoside complex (New England Biolabs) using a tissue lyser (Qiagen). The lysate was centrifuged at 10,000 × *g* for 10 min at 4 °C. The supernatant was applied to a linear 20%–47% sucrose gradient in 20 mm Tris-HCl (pH 8.0), 140 mm KCl, and 5 mm MgCl_2_ and subjected to centrifugation at 40,000 rpm for 3 h with Beckman SW41 rotors. 1-ml fractions were collected with concomitant measurement of the absorbance at 260 nm. RNAs were recovered from polysome-containing fractions by extraction with an equal volume of phenol–chloroform–isoamyl alcohol and analyzed as in Ref. 4.

### mRNA turnover

AML12 cells were treated with actinomycin D (1 mm, Sigma). At the indicated times, the total RNAs were extracted, followed by DNase digestion and then complementary DNA synthesis. The amount of mRNA was quantified by real-time PCR.

## Author contributions

L. R., J. A., and N. D. conceptualization; L. R. and N. D. investigation; L. R. methodology; L. R., L. M., J. A., and N. D. writing-review and editing; L. M. and J. A. resources; J. A. and N. D. supervision; J. A. and N. D. writing-original draft; J. A. project administration.

## Supplementary Material

Supporting Information

## Footnotes

4 The abbreviations used are:

IMP: IGF2 mRNA binding protein
E10.5: embryonic day 10.5
HFD: high-fat diet
ASM: acid-soluble metabolite(s)
IP: immunoprecipitate
QPCR: quantitative PCR.
