# Supplementary material for: Liver-specific deletion of IGF2 mRNA binding protein-2/IMP2 reduces hepatic fatty acid oxidation and increases hepatic triglyceride accumulation
Source: J Biol Chem. 2019 Jun 17;294(31):11944–51. doi: 10.1074/jbc.RA119.008778 (PMC6682725; doi:10.1074/jbc.RA119.008778)
Supplement: Supporting Information [file supp_294_31_11944__index.html]

Liver-specific deletion of IGF2 mRNA binding protein-2/IMP2 reduces hepatic fatty acid oxidation and increases hepatic triglyceride accumulation — hepatocyte IMP2 stabilizes CPT1A and PPARα mRNAs — Liver-specific deletion of IGF2 mRNA binding protein-2/IMP2 reduces hepatic fatty acid oxidation and increases hepatic triglyceride accumulation — Hepatocyte IMP2 stabilizes CPT1A and PPARα mRNAs — Supporting Information 

# Liver-specific deletion of IGF2 mRNA binding protein-2/IMP2 reduces hepatic fatty acid oxidation and increases hepatic triglyceride accumulation

## Supporting Information

- Supporting Information (to be published online) - Figure S1-S3+legend+Table S1
