# Supplementary material for: Liver-specific deletion of IGF2 mRNA binding protein-2/IMP2 reduces hepatic fatty acid oxidation and increases hepatic triglyceride accumulation
Source: J Biol Chem. 2019 Jun 17;294(31):11944–51. doi: 10.1074/jbc.RA119.008778 (PMC6682725; doi:10.1074/jbc.RA119.008778)
Supplement: Supporting Information [file supp_RA119.008778_144770_2_supp_343299_psn0pw.pdf]

Supporting information-Reguè et.al.

Figure S1. Body weight (A) and body composition (B) of male *Imp2ff* and *Imp2ff*/Alb-Cre$^{+/-}$ mice fed normal chow (NCD or a high fat diet (HFD). Body composition was determined using ECHO-MRI.

Figure S2. Liver triglyceride levels in *Imp2ff* and *Imp2ff*/Alb-Cre$^{+/-}$ mice. **A.** Males, 5 pairs, 10 weeks old, HFD from 4 weeks age. **B.** Females, 4 pairs, 30 weeks old, normal chow only. **C.** Females, 6 Imp2ff, 8 *Imp2ff*/Alb-Cre$^{+/-}$, HFD from 4 weeks age. ns=p>0.05.

Figure S3. Liver histology in *Imp2ff* and *Imp2ff*/Alb-Cre$^{+/-}$ mice on a high fat diet. Liver was excised at 30 weeks age from male mice, fixed and stained with H&E. Representative sections from two pairs are shown. Scale bar=150μM.

Table S1. Antibody sources and oligonucleotides for QPCR.

Antibodies

PPARα	Santa Cruz	sc-398394
PPARγ	Cell Signaling	cst-2430
RPL26	Sigma	SAB2107642
CPT1A	Abcam	ab128568
CPT2	Abcam	ab110293

Primer List

Acadl	AGAAGTTCATCCCCCAGATGAC	GGCGTTCGTTCTTACTCCTTGT
Acc1	GCGGCTACAGGGACTATACTG	CGGAAGTAAGAGCTACTAGCGG
Acc2	GGGCTCCCTGGATGACAAC	GCTCTTCCGGGAGGAGTTCT
Acly	AAGAAGGAGGGGAAGCTGAT	TCGCATGTCTGGGTTGTTTA
Acot7	GAGGGTCGGAAACGCTATGAA	GGCTGGACAATGTCTCCGTTC
Agpat1	GCTGGCTGGCAGGAATCAT	GTCTGAGCCACCTCGGACAT
Angptl4	TCCAACGCCACCCACTTAC	TGAAGTCATCTCACAGTTGACCA
Apoa4	CCAAGATCGACCAGAACGTGG	GTCCTGAGCATAGGGAGCCA
ApoB	TGGGATTCCATCTGCCATCTCGAG	GTACAGATCCATCACAGGACAATG
Apoc3	GCGTGCAGGAGTCCGATATAG	CCAGTAGCCTTTCAGGGATCT
cd36	GCTTGCAACTGTCAGCACAT	GCCTTGCTGTAGCCAAGAAC
Chrebp	CCTTCGCCAACTCAGCACTT	TGGCTTGCTCAGGCACAA
Cpt1a	CTATGCGCTACTCGCTGAAGG	GGCTTTCGACCCGAGAAGA
Cpt2	GCTCCGAGGCATTTGTC	CATCGCTGCTTCTTTGGT
Dgat1	CAGACCAGCGTGGGCG	GAACAAAGAGTCTTGCAGACGATG
Eci2	CCCTTCTGGGACTATTTGATGC	CTTCGGAAACGTGTAAGAGGAG
Fabp1	GGAATTGGGAGTAGGAAGAGCC	TGGACTTGAACCAAGGAGTCAT
Fabp4	AAGGTGAAGAGCATCATAACCCT	TCACGCCTTTCATAACACATTCC
Fabp5	AAAGAGCTAGGAGTAGGACTGG	TGTTGCCATCACACGTAATGA
Fads2	ATTCGGGAGAAGATGCTACG	AAGAACTTGCCCACGAAGTC
Fas	GCGATGAAGAGCATGGTTTAG	GGCTCAAGGGTTCCATGTT
Fgf21	AAAGCCTCTAGGTTTCTTTGCCA	CCTCAGGATCAAAGTGAGGCG
GAPDH	CTGGAGAAACCTGCCAAGTA	TGTTGCTGTAGCCGTATTCA
Gpat	ACGCACACAAGGCACAGAG	TGCTGCTCAGTACATTCTCAGTA
Imp2	GACTACCCCGACCAGAACTG	GAGGCGGGATGTTCCGAATC
Lipin 1	GAGCATGCCAAGACCAACATC	CAATGGGAAGACGTGATCGA
Lpl	ACTCTGTGTCTAACTGCCACTTCAA	ATACATTCCCGTTACCGTCCAT
Mtp	TATGGAGATCCAGGGTGGTC	CTGCTTTCCACACCAGCTTT
Pparα	TCGAGGAAGGCACTACACCT	TCTTCCCAAAGCTCCTTCAA
Pparγ	CGCTGATGCACTGCCTATGA	AGAGGTCCACAGAGCTGATTCC
scd1	CTGTACGGGATCATACTGGTTC	GCCGTGCCTTGTAAGTTCTG
Screbp2	AGCCAAGGAGAGCCTGTACTG	GAGAGCGCACAGCTGCATCG
Srebp1c	GGAGCCATGGATTGCACATT	GGCCCGGGAAGTCACTGT
18s rRNA	GTAACCCGTTGAACCCCATT	CCATCCAATCGGTAGTAGCG

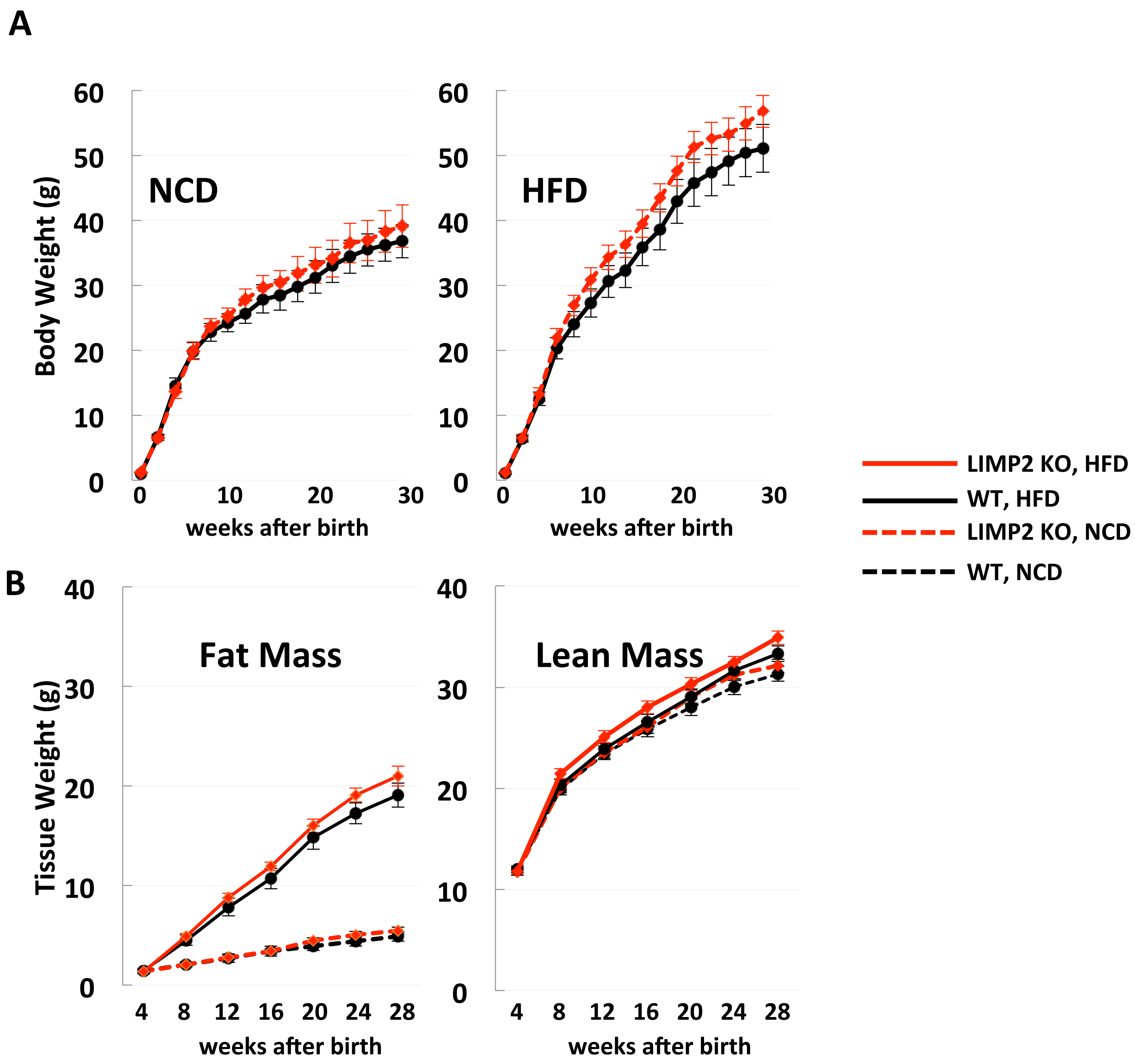
A
NCD
HFD
Body Weight (g)
60
50
40
30
20
10
0
0
10
20
30
weeks after birth
B
Fat Mass
Lean Mass
Tissue Weight (g)
40
30
20
10
0
4
8
12
16
20
24
28
weeks after birth
LIMP2 KO, HFD
WT, HFD
LIMP2 KO, NCD
WT, NCD

Fig. S1

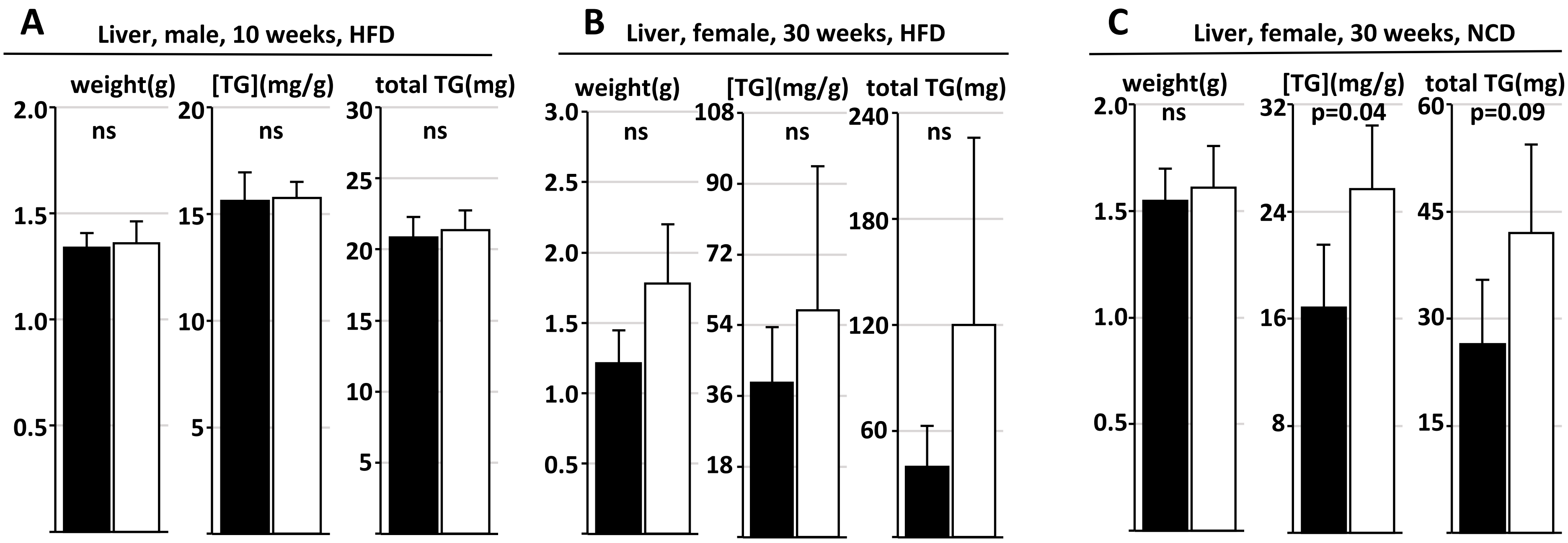
A
Liver, male, 10 weeks, HFD
weight(g)
[TG](mg/g)
total TG(mg)
ns
ns
ns
B
Liver, female, 30 weeks, HFD
weight(g)
[TG](mg/g)
total TG(mg)
ns
ns
ns
C
Liver, female, 30 weeks, NCD
weight(g)
[TG](mg/g)
total TG(mg)
ns
p=0.04
p=0.09

Fig. S2

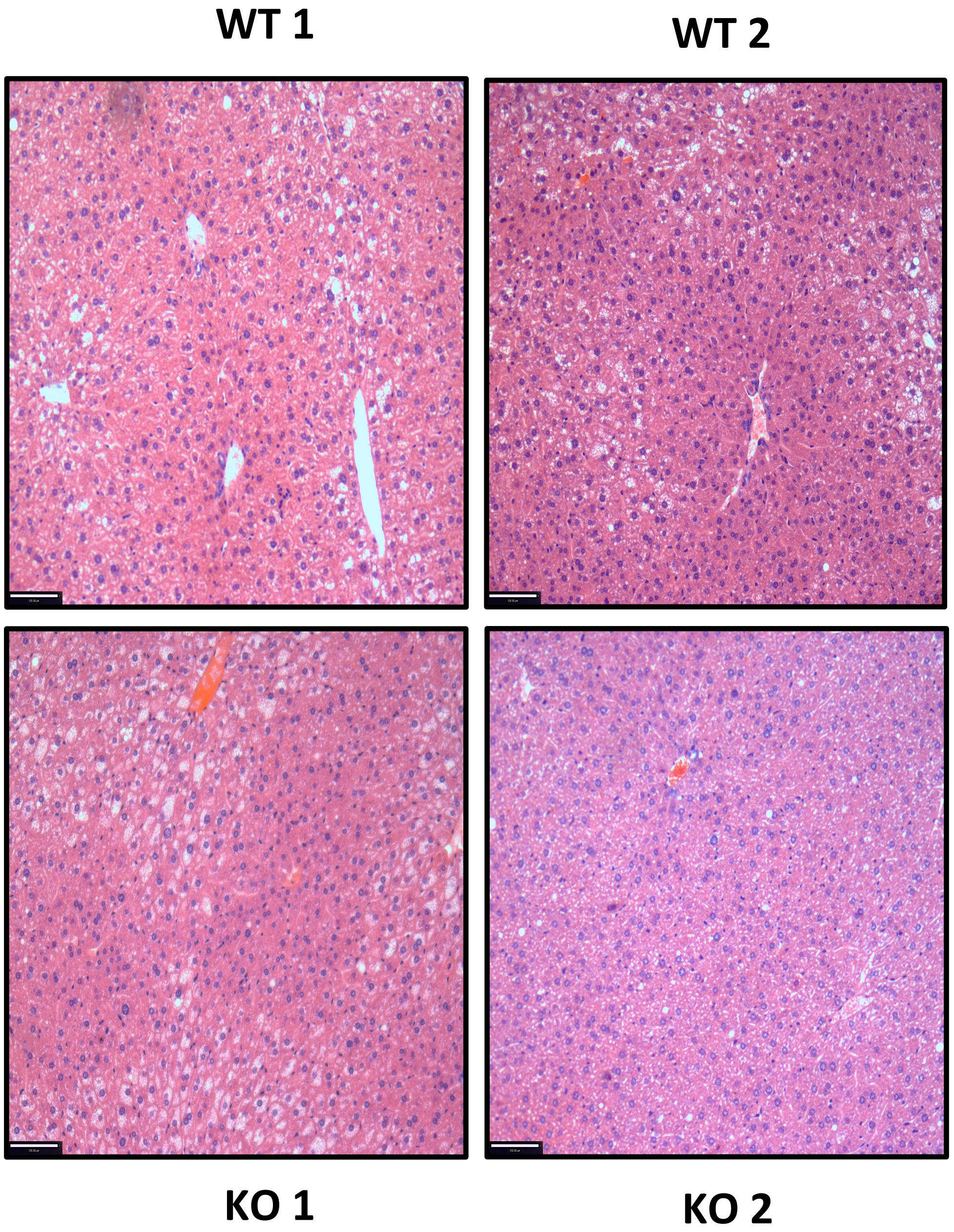

Fig. S3
